# Modulation of Transient Receptor Potential C Channel Activity by Cholesterol

**DOI:** 10.3389/fphar.2019.01487

**Published:** 2019-12-13

**Authors:** Rita Gutorov, Maximilian Peters, Ben Katz, Tal Brandwine, Nicolas A. Barbera, Irena Levitan, Baruch Minke

**Affiliations:** ^1^Institute for Medical Research Israel-Canada (IMRIC), Edmond and Lily Safra Center for Brain Sciences (ELSC), Faculty of Medicine, The Hebrew University, Jerusalem, Israel; ^2^Division of Pulmonary, Critical Care, Sleep and Allergy, Department of Medicine, University of Illinois at Chicago, Chicago, IL, United States

**Keywords:** TRP-like (TRPL) channel, lipid rafts, methyl-β-cyclodextrin, cholesterol recognition amino acid consensus sequence (CRAC), caveolae

## Abstract

Changes of cholesterol level in the plasma membrane of cells have been shown to modulate ion channel function. The proposed mechanisms underlying these modulations include association of cholesterol to a single binding site at a single channel conformation, association to a highly flexible cholesterol binding site adopting multiple poses, and perturbation of lipid rafts. These perturbations have been shown to induce reversible targeting of mammalian transient receptor potential C (TRPC) channels to the cholesterol-rich membrane environment of lipid rafts. Thus, the observed inhibition of TRPC channels by methyl-β-cyclodextrin (MβCD), which induces cholesterol efflux from the plasma membrane, may result from disruption of lipid rafts. This perturbation was also shown to disrupt multimolecular signaling complexes containing TRPC channels. The *Drosophila* TRP and TRP-like (TRPL) channels belong to the TRPC channel subfamily. When the *Drosophila* TRPL channel was expressed in S2 or HEK293 cells and perfused with MβCD, the TRPL current was abolished in less than 100 s, fitting well the fast kinetic phase of cholesterol sequestration experiments in cells. It was thus suggested that the fast kinetics of TRPL channel suppression by MβCD arise from disruption of lipid rafts. Accordingly, lipid raft perturbation by cholesterol sequestration could give clues to the function of lipid environment in TRPC channel activity and its mechanism.

## Introduction

Cholesterol molecules are intercalated among the phospholipids of cell membrane forming an integral part of the plasma membrane, which is essential for the proper function of ion channels. Plasma membrane cholesterol includes domains known as lipid rafts (Pike, 2006). However, cholesterol is located in both rafts and non-raft fractions.

An efficient method to modulate the content of plasma membrane cholesterol is by methyl-β-cyclodextrin (MβCD), which is a cyclic oligosaccharide (Ohtani et al., 1989; Davis and Brewster, 2004). The β-cyclodextrins (seven glucose units) have high affinity for encapsulating cholesterol (Ohtani et al., 1989). MβCD is quite specific for cholesterol, allowing enrichment or a relatively rapid sequestration of cholesterol from living cells. Cholesterol-saturated MβCD is efficient as cholesterol donor. The degree of cholesterol enrichment is between ∼30% to ∼threefold, according to the type of cell (Christian et al., 1997; Levitan et al., 2000). When cells are incubated with high concentration of “empty” MβCD (5–10 mM) for hours (> 2 h), 80–90% of total cellular cholesterol can be sequestered (Kilsdonk et al., 1995; Levitan et al., 2000). The amount of cholesterol sequestration from different cell types is a highly variable parameter (Matthews et al., 1985; Kilsdonk et al., 1995; Christian et al., 1997; Niu et al., 2002). Cholesterol sequestration leads to dis-association of proteins from lipid rafts (Scheiffele et al., 1997; Kabouridis et al., 2000; Predescu et al., 2005) and to decrease in clustering of raft-associated molecules (Harder et al., 1998). Cholesterol depletion also disrupts caveolae structure; it does not result in the disappearance of caveolin but leads to a shift of caveolin from raft to non-raft fractions (Hissa et al., 2017) and to ruffling (Grimmer et al., 2002). It was shown that βCDs sequestered cholesterol from both cholesterol-rich and cholesterol-poor membrane domains (Ottico et al., 2003; Gaus et al., 2005; Rouquette-Jazdanian et al., 2006; Tikku et al., 2007).

In this mini-review, we discuss physiological effects of modulating plasma membrane cholesterol. We focus on modulations of transient receptor potential C (TRPC) channels activity following application MβCD, with emphasis on fast modulations (in less than 100 s).

## The Kinetics of Cholesterol Removal by MβCD

Cholesterol sequestration by βCD from several cell types revealed bi-exponential kinetics, when monitored with radiolabeled [H^3^]cholesterol: a fast (*τ* 1/2 of 19–23 s) and a slow (*τ* 1/2 of 15–30 min) kinetics, suggesting the existence of two separate pools of cholesterol (Yancey et al., 1996). It was further suggested that the “fast” pool of cholesterol corresponds to the outer leaflet of the plasma membrane, while the identity of the slow pool was unclear (Yancey et al., 1996). Recently, imaging studies showed that cholesterol level in the inner leaflet of the plasma membrane was ∼12-fold smaller than cholesterol concentration in the outer leaflet (Liu et al., 2017). Interestingly, two pools of MβCD extracts were observed, with half-lives similar to those reported previously (Yancey et al., 1996; Haynes et al., 2000). However, it was also found that the slower cholesterol efflux (from the “slow” pool) was absent from energy-depleted cells (Hao et al., 2002).

The existence of a fast-modulated pool of cholesterol is important for the interpretation of studies showing fast responses of ion channels to modulations of cholesterol (see below). Unfortunately, most studies used prolonged incubations of cells and tissues with cyclodextrins, thus precluding the ability to observe fast kinetics of cholesterol modulations.

## Modulation of Mammalian TRPC Channel Activity by Cholesterol

Cholesterol-ion channels interactions have been studied both computationally and experimentally. Earlier studies identified two types of cholesterol binding motifs: the cholesterol consensus motif (CCM) and the cholesterol recognition amino acid consensus sequence (CRAC), as well as the so-called CARC motif, in which the amino acid sequence appears in reverse. These motifs have been found in many ion channels, such as nicotinic acetylcholine receptor (nAChR), BK, P2X7, Kv1.3, as well as TRPV1 channels (Picazo-Juárez et al., 2011; Singh et al., 2012; Balajthy et al., 2017; Murrell-Lagnado, 2017). However, a recent analysis of the solved crystal structures of 24 cholesterol–protein complexes with 38 distinct cholesterol binding sites showed that fewer than 40% of these sites contained a CRAC or CARC motif, indicating that these motifs at best form only a subset of potential cholesterol binding sites (Rosenhouse-Dantsker, 2017). Furthermore, the relatively loose definition of the motif, (L/V)-X_1–5_-(Y)-X_1–5_-(K/R), where X can be one to five residues of any kind, has raised concerns about the predictive nature of the motif and the risk of identifying false positives (Epand, 2006; Jaipuria et al., 2018). Notably, while for many channels, there is little or no experimental confirmation for cholesterol interacting with these motifs, it was shown that a CRAC motif has a significant effect on cholesterol modulation of TRPV1 channel activity (Picazo-Juárez et al., 2011).

**Figure 1:**
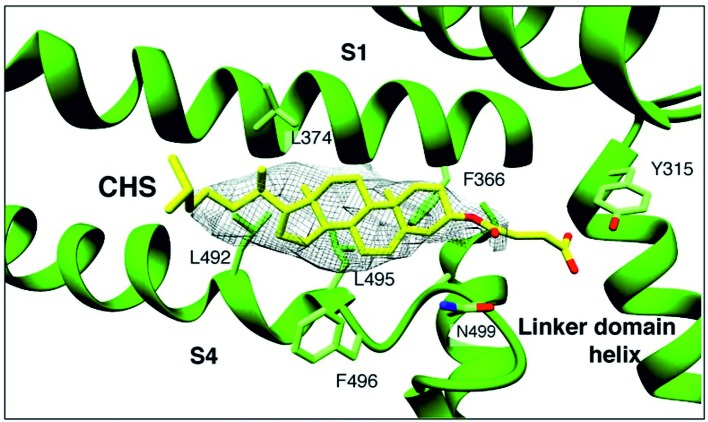
A sterol binding pocket in the TRPC4_DR_ structure. Electron cryo-microscopy structure of zebra fish TRPC4 (TRPC4_DR_) channel in its unliganded closed state, at an overall resolution of 3.6 Å. The transmembrane S1–S6 helices structure revealed that in the pre-S1 elbow domain inside the membrane, a cavity is formed with helices S1 and S4, in which a density corresponding to a sterol is formed. (Reproduced from Vinayagam et al. (2018) with permission from eLife.)

Because the above cholesterol binding motifs represent only a subset of potential cholesterol binding sites, there is a risk in using this approach. Consequently, more recent strategies for identifying binding sites have utilized computational approaches such as docking analyses and molecular dynamics simulations to identify potential sites in an unbiased manner. In particular, cholesterol binding sites were identified on nAChR, as well as on inwardly rectifying potassium (Kir) channels, voltage-dependent anion channel (VDAC), and GABA_A_ receptors (Brannigan et al., 2008; Rosenhouse-Dantsker et al., 2013; Hénin et al., 2014; Weiser et al., 2014). In these studies, docking analyses were first used to predict a set of candidate binding sites, which were then refined through short atomistic simulations and tested experimentally. Importantly, these binding sites did not contain the previously described cholesterol binding motifs. A limitation of this approach, however, is that atomistic simulations usually are not long enough to observe the dynamic behavior of the cholesterol molecule moving from the bilayer to the binding site. This limitation is addressed most recently, with the development of coarse-grained force fields such as the Martini force field, long (µs) time-scale simulations of membrane proteins allowing for the dynamic binding and unbinding of cholesterol to target proteins, providing deeper insight into the mechanisms of cholesterol regulation (Cang et al., 2013; Genheden et al., 2017; Rouviere et al., 2017; Barbera et al., 2018). Using these approaches, most recent studies discovered that in contrast to most other ligands, cholesterol binding is highly flexible and cholesterol dynamically explores its binding site, adopting multiple poses in a “cloud,” rather than occupying a single conformation (Gimpl, 2016; Genheden et al., 2017; Rouviere et al., 2017; Barbera et al., 2018). Recently electron cryo-microscopy structure of zebra fish TRPC4 (TRPC4_DR_) channel in its unliganded closed state, at an overall resolution of 3.6 Å was published (Vinayagam et al., 2018). The transmembrane S1–S6 helices structure revealed that in the pre-S1 elbow domain inside the membrane, a cavity is formed with helices S1 and S4, in which a density corresponding to a sterol is formed (**Figure 1**). Since the authors added cholesteryl hemisuccinate (CHS) during the purification of TRPC4_DR_, they fitted this molecule into the density. This density in the S1–S4 cavity is consistent with the above notion that sterol binding to channel proteins is flexible.

Cholesterol changes in the plasma membrane of cells have been shown to modulate ion channels function, and these modulations include perturbation of specific lipid environments. Experimental observations have suggested reversible targeting of mammalian TRPC channels to cholesterol-rich membrane environment of lipid rafts. This led to the suggestion that the observed inhibition of mammalian TRPC channels by MβCD-inducing cholesterol efflux may result in part from disruption of lipid rafts, including disruption of multimolecular signaling complexes (Svobodova and Groschner, 2016). Below we give examples for the role of lipid rafts in several types of TRPC channels.

## TRPC1

The mammalian TRPC1 was shown to interact with caveolin-1 (Cav-1), which is a scaffolding protein that binds cholesterol (Lockwich et al., 2000). This interaction is mediated *via* both N-terminal Cav-1 binding motif and C-terminal Cav-scaffolding consensus. The important role of caveolae in TRPC1 activation was supported by the finding that TRPC1 activity was dependent on Cav-1 (Murata et al., 2007), while TRPC1 was found mainly in caveolae (Lockwich et al., 2000). The experiments indicated that TRPC1 mainly resides in lipid rafts and exposed to the cholesterol-rich membrane of caveolae (Lockwich et al., 2000). Inhibition of TRPC1 currents by MβCD-induced cholesterol sequestration may result from both disruption of lipid raft architecture, including impaired local assembly of signaling molecules, and inhibition of a gating mechanism. The above two effects of cholesterol on TRPC1 function (i.e. disruption of lipid raft architecture and inhibition of a gating mechanism) was demonstrated for several cell types (Bergdahl et al., 2003; Brownlow and Sage, 2005; Kannan et al., 2007).

## TRPC3

Cholesterol sensitivity of the TRPC3 channel was demonstrated by using acute administration of cholesterol-saturated MβCD to modify membrane cholesterol content. Cholesterol application elevated conductance in TRPC3-expressing HEK293 cell culture. The membrane conductance derived from I–V curves was typical for phospholipase C (PLC)–mediated TRPC3 currents, showing fast rise time of several seconds. Thus, increased cholesterol concentrations induced a relatively fast TRPC3-mediated current in HEK293 cells (Graziani et al., 2006). Surface biotinylation experiments revealed a significant increase of TRPC3 level at the plasma membrane caused by cholesterol addition. This result suggests that TRPC3-mediated current and the ensuing Ca^2+^ influx that were induced by cholesterol elevation may resulted from a cholesterol-induced expression of TRPC3 in the surface membrane (Graziani et al., 2006).

Like TRPC1, the TRPC3 channels reside in caveolae. The significance of caveolae in mediating inositol 1,4,5 trisphosphate (IP_3_)–induced non-selective cation current (I_Cat_) activation and arterial smooth muscle constriction was studied in smooth muscles of cerebral arteries (Adebiyi et al., 2011). Immunoprecipitation and immunoFRET experiments revealed that Cav-1, TRPC3, and IP_3_ receptor1 (IP_3_R1-1) formed a multimolecular signaling complex *via* Cav-1 scaffolding domain that was reversibly disrupted by MβCD and by a peptide with Cav-1 scaffolding domain. These experiments revealed close association of the signaling proteins in smooth muscles of cerebral arteries. In other experiments, caveolae disassembly was obtained by: (1) MβCD, (2) Cav-1 knockdown using RNAi, or (3) application of Cav-1 scaffolding domain. Caveolae disassembly inhibited the I_Cat_ currents and vasoconstriction. The data thus indicated that the multimolecular signaling complex *via* Cav-1 scaffolding domain allowed signal-induced vasoconstriction (Adebiyi et al., 2011).

## TRPC6

Podocytes are multipolar cells that cover the external surface of glomerular capillaries and form an essential component of the kidney ultrafiltration apparatus (Pavenstädt et al., 2003). Importantly, in order for podocytes to respond to distending forces, their Ca^2+^-dependent contractile elements must be coupled to a Ca^2+^ signaling pathway. The major source of regulated Ca^2+^ influx into podocytes is Ca^2+^-permeable TRPC6 channel, which like other mammalian TRPC channels are activated *via* PLC-mediated signaling cascade (Dryer and Reiser, 2010; Katz et al., 2017). Several mutations in the TRPC6 channel result in autosomal-dominant nephrotic syndromes (Reiser et al., 2005; Winn et al., 2005; Heeringa et al., 2009). Podocin is a cholesterol binding protein, which interacts, in a still unclear manner, with the TRPC6 channel (Reiser et al., 2005; Huber et al., 2006). The ability of podocin, which resides in lipid raft domains (Lei et al., 2014), to bind cholesterol may be central to TRPC6 gating (Huber et al., 2006). Inhibition of TRPC6 channels by application of MβCD or by expression of dominant-negative Cav-1 isoform indicated that TRPC6 activation requires lipid rafts regions at the surface membrane (Lei et al., 2014), suggesting that podocin coordinates TRPC6 channel activity (Anderson et al., 2013).

## Fast Inhibition of the *Drosophila* Trp-Like Channel Activity by MβCD

The *Drosophila* light-sensitive TRP and TRP-like (TRPL) channels are the first members of the TRPC subfamily that were discovered (review Katz et al., 2017). It is well established that the TRP/TRPL channels are the target of the rhodopsin-activated-phosphoinositide cascade, which leads to production of lipids that may function as second messenger in a variety of cells and tissues. Cyclodextrins, both α-cyclodextrin and MβCD, are known to sequester phospholipids (Ohtani et al., 1989) that are involved directly or indirectly in gating of TRPC channels.

**Figure 2:**
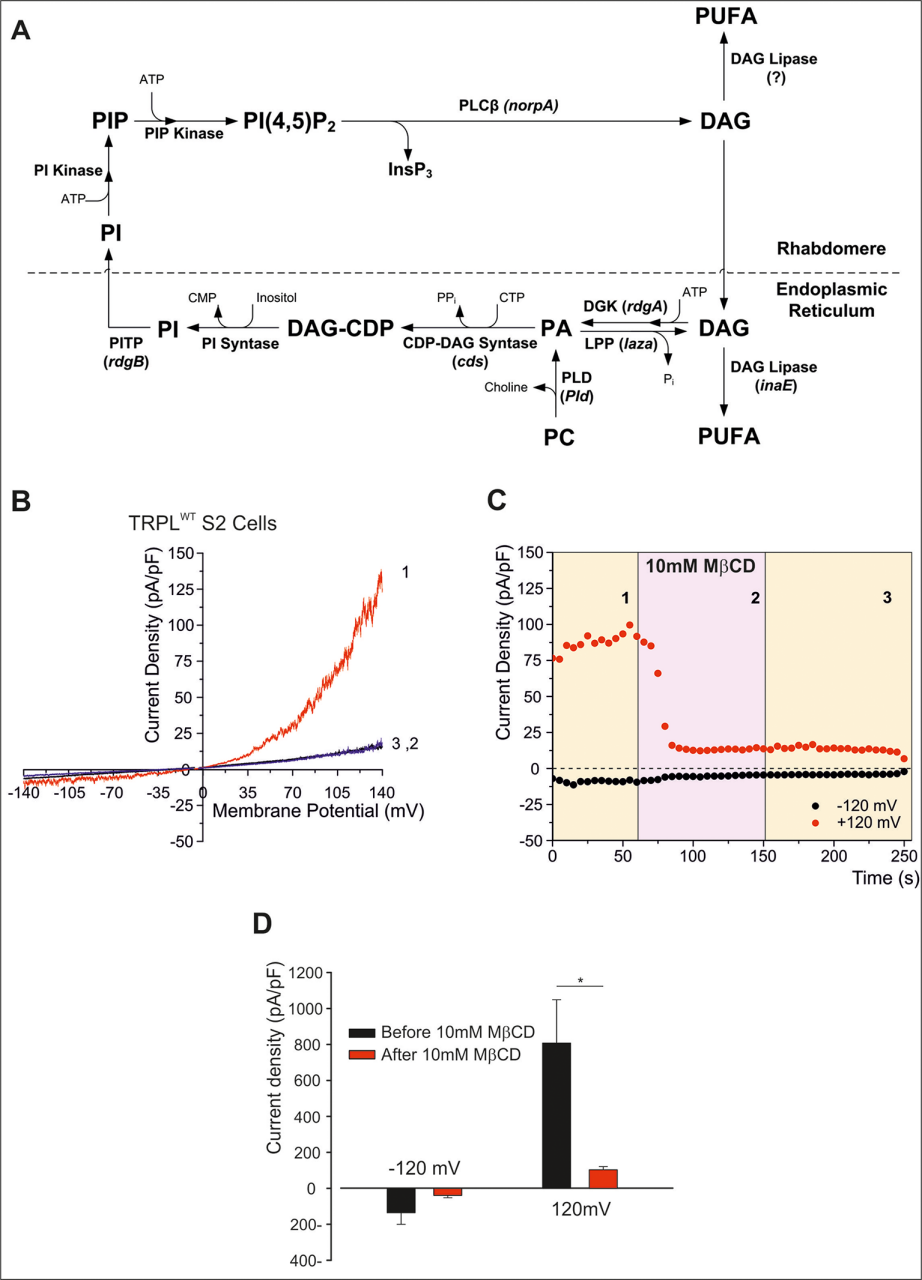
**(A)** *The phosphoinosite (PI) cycle*. In the phototransduction cascade, light triggers the activation of phospholipase Cβ (PLCβ, encoded by *norpA*). This catalyzes hydrolysis of the membrane phospholipid PI(4,5)P_2_ (PIP_2_) into IP_3_ and diacylglycerol (DAG). DAG is transported by endocytosis to the endoplasmic reticulum and inactivated by phosphorylation converting it into phosphatidic acid (PA) *via* DAG kinase (DGK, encoded by *rdgA*) and to CDP-DAG *via* CDP-DAG synthase. Subsequently, CDP-DAG is converted into phosphatidyl inositol (PI), which is transferred back to the microvillar membrane, by the PI transfer protein (encoded by *rdgB*). PIP and PIP_2_ are produced at the microvillar membrane by PI kinase and PIP kinase, respectively. PA can also be converted back to DAG by lipid phosphate phosphohydrolase (Lpp, encoded by laza). PA is also produced from phosphatidyl choline (PC) by phospholipase D (PLD). DAG is also converted in two enzymatic stages, one of them is by DAG lipase (encoded by *inaE*), into polyunsaturated fatty acids (PUFAs). **(B**–**D)** *MβCD blocks constitutive TRPL channels activity*. **(B)** Current–voltage relationships (I–V curves) measuring TRPL-dependent currents. I–V curves obtained in response to voltage ramp (of 1 s duration) from S2 cells expressing TRPL and showing basal channel activity with strong outward rectification, typical for TRPL-dependent current (1). The TRPL channel activity was highly reduced after perfusion with 10 mM methyl-β-cyclodextrin (MβCD) (2) and the effect was irreversible, even after washout of MβCD (3) (n > 10). **(C)** Time course of the MβCD effects on TRPL currents in S2 cells. Current densities are shown as a function of time. Series of I–V curves were derived from repeatedly applied voltage ramps every 5 s, and currents were measured at ±120 mV holding potentials as a function of time under the various experimental conditions as indicated. The numbers correspond to the numbers on the I–V curves in **(B)**. **(D)** Statistics of the cholesterol depletion experiments in S2 cells. **(A)** Cholesterol depletion by MβCD had a significant effect on the positive TRPL currents at 120 mV (n = 5, values are average ± SEM, paired Student t-test, *p ≤ 0.05). Reproduced from Katz and Minke, 2009 with permission from Frontiers. **(B**–**D)** Reproduced from Peters et al., 2017 with permission from Elsevier, license number 4676401165468.)

Light activation of PLC in *Drosophila* photoreceptors leads to the formation of diacylglycerol (DAG) and IP_3_, which are then recycled back to form *phosphatidylinositol 4*,*5*-*bisphosphate* [PtdIns(4,5)P_2_, designated the phosphoinositide (PI) cycle, **Figure 2A**). The mechanism by which the PI cycle activates the TRP/TRPL channels is not entirely clear (e.g. see Hardie, 2003). Nevertheless, the involvement of lipids in TRP/TRPL channel activation may account for the effects of MβCD on the TRPL channel (see below).

To characterize the *Drosophila* light-sensitive channels, TRPL channels were expressed in tissue-cultured S2 (Hardie et al., 1997; Chyb et al., 1999; Parnas et al., 2009; Lev et al., 2012b; Peters et al., 2017) and HEK293 cells (Hambrecht et al., 2000; Lev et al., 2012b; Peters et al., 2017). TRPL channels expressed in the *Drosophila* S2 cells showed basal activity that could be amplified by polyunsaturated fatty acid [PUFA, e.g. linoleic acid (LA), Lev and Minke, 2010; Lev et al., 2012a; Lev et al., 2012b]. The pronounced basal TRPL current obtained at positive voltage was virtually abolished by MβCD, in less than 100 s (**Figures 2B–D**). This quick effect of cholesterol sequestration is in marked contrast to the previously shown slow effect (of many minutes) of cholesterol sequestration (Singh et al., 2011). Inhibition of the TRPL current persisted long after removal of MβCD, excluding direct inhibition of TRPL by MβCD (**Figure 2C**). In further experiments, cholesterol was first depleted by means of MβCD in S2 cells expressing TRPL; then the excess of MβCD was washed out, and the cells were perfused with the TRPL channel activator LA. This protocol initially resulted, as expected, in reduction of TRPL currents (see **Figure 2**), but surprisingly, LA could activate the TRPL channels independent of MβCD. These results suggest that in S2 cells, MβCD does not affects the TRPL channels directly but affected G-protein coupled related signaling proteins upstream of TRPL in the cascade. Also, it is possible that cholesterol and LA share common mechanism of action, or that LA activation does not require cholesterol for its action.

**Figure 3:**
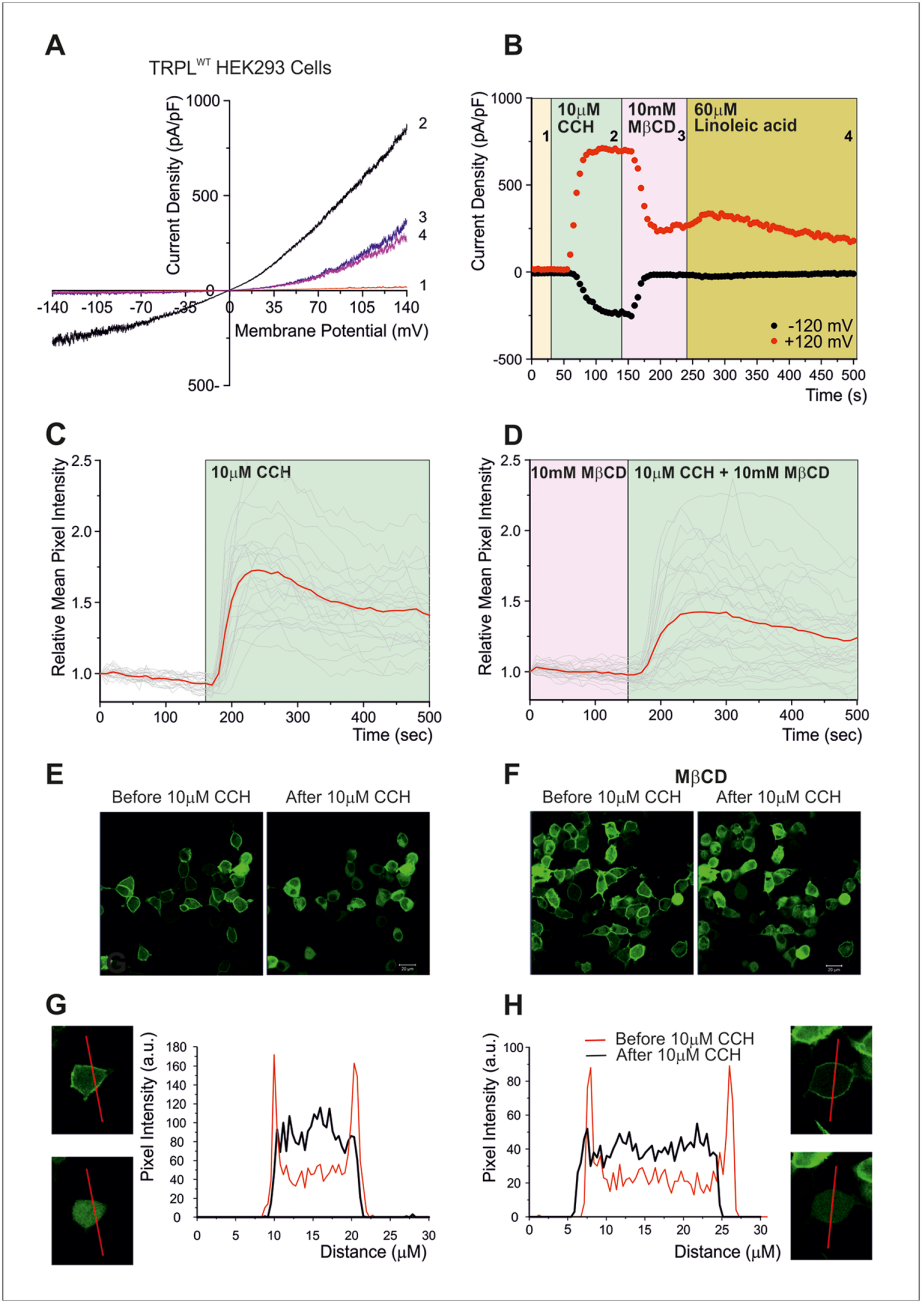
**(A**–**B)** *Cholesterol depletion suppressed receptor-activated TRPL-dependent current*. **(A)** TRP-like–green fluorescent protein (TRPL–GFP) did not show any spontaneous activity in HEK293 cells, but it could be readily activated *via* PLC and blocked by MβCD: current–voltage relationships measured from HEK293 cells expressing TRPL–GFP, showing no basal channel activity (1). However, coexpression of the hM1 muscarinic receptor and application of carbachol (CCH) activated the expressed TRPL–GFP channels *via* endogenous PLC-mediated cascade (2) and the TRPL-dependent current in the presence of CCH was suppressed by application of MβCD (3), while subsequent application of LA, a strong activator of TRPL channels, did not activate the channels after the application of MβCD (4). **(B)** Time course of the receptor-activated TRPL-dependent current and the effect of cholesterol depletion on the receptor-activated TRPL currents in HEK293 cells. Current densities are shown as a function of time. Series of i–V curves were derived from repeatedly applied voltage ramps every 5 s, and currents were measured at ±120 mV holding potentials as a function of time under the various experimental conditions as indicated **(C**–**H)** *Cholesterol depletion did not affect receptor-induced PLC activity*. No effects of cholesterol depletion on PLC activity as monitored by translocation of the PIP_2_ sensor PH–GFP: representative series of multiphoton images of HEK293 cells coexpressing eGFP-tagged PH domain and hM1 receptor. Application of CCH to the bathing solution, in a concentration that activated the TRPL channels (10 μM CCH), induced similar translocation of the eGFP-tagged PH domain to the cell body, with and without MβCD, indicating the PLC-mediated hydrolysis of PIP_2_ is not affected by MβCD. **(C)** The time course of fluorescence changes measured in the cytosol before application of MβCD: graph plotting the relative mean pixels’ intensity (red curve) as a function of time measured from multiphoton images of HEK293 cells expressing PH–GFP and hM1 receptors. Before PLC stimulation by CCH application (white background), the GFP–PH is associated with the plasma membrane where most PIP_2_ is located and the cell body fluorescence is low (for quantification, see **G**, **H**). Once PLC is activated and PIP_2_ is hydrolyzed (green background), the PH–GFP translocates to the cytosol and there is a marked increase in fluorescence intensity at the cytosol. The individual single-cell measurements are shown by noisy dim gray traces. **(D)** The time course of fluorescence changes measured in the cytosol after application of MβCD: similar graph as in **(C)**, but measured following application of MβCD. **(E)** Multiphoton images of HEK293 cells expressing PH–GFP and hM1 receptor without application of MβCD: Left: GFP fluorescence of cells before application of CCH, little PH–GFP translocation was observed. Right: GFP fluorescence of cells after perfusion with the M1 agonist CCH. Translocation of PH–GFP is observed. MβCD was not applied (n > 50). **(F)** Multiphoton images of HEK293 cells expressing PH–GFP and hM1 receptor after application of MβCD: Similar images of HEK293 cells expressing TRPL PH–GFP and hM1 receptor before (left) and after application of CCH (right). MβCD was applied, but it did not affect PH–GFP translocation (n > 50). **(G**, **H)** Graphs plotting the PH–GFP fluorescence intensity as a function of cell position: fluorescence intensity of images showing cross sections of two representative cells along the red line, before application of CCH (red curve), and after application of CCH (black curve) in the absence of MβCD **(G)** and after application of MβCD **(H)**. (Reproduced from Peters et al., 2017 with permission from Elsevier, license number 4676401165468.)

To further examine at what stage of the transduction cascade MβCD operates, we expressed the Pleckstrin Homology domain of PLC-δ attached to the green fluorescent protein (GFP) in tissue culture cells. The Pleckstrin Homology domain, which binds specifically PtdIns(4,5)P_2_ (and IP_3_), marks plasma membrane PtdIns(4,5)P_2_ in living cells (Balla and Varnai, 2002; Suh et al., 2006; Lev et al., 2012b). Since it is technically difficult to perform these experiments in S2 cells, HEK293 cells were used. In HEK293 cells expressing TRPL, no basal (constitutive) TRPL current was observed [(**Figure 3A**), unlike the situation in S2 cells (**Figures 2B, C**, Lev et al., 2012a)]. Under control conditions, the Pleckstrin Homology domain–GFP was associated with PtdIns(4,5)P_2_ of the surface membrane (**Figures 3C–H**). However, when PtdIns(4,5)P_2_ concentrations were reduced [e.g. by PLC-dependent hydrolysis of PtdIns(4,5)P_2_], the GFP-associated peptide diffused to the cell body (**Figures 3C–H**). PLC was activated by the expressed muscarinic receptor (hM1, which was activated by carbachol, CCH, **Figures 3C, D**), before (**Figure 3C**) and after application of MβCD (**Figure 3D**). These experiments showed that MβCD had no effect on PtdIns(4,5)P_2_ hydrolysis by PLC, thus indicating that inhibition of TRPL-dependent current by cholesterol sequestration takes place after activation of PLC in the transduction cascade in HEK293 cells. Strikingly, application of LA, which is a highly potent activator of the TRPL channels, acting directly on the channels (Parnas et al., 2009) had virtually no effect (**Figure 3B**). Thus, in HEK293 cells, LA applied after response suppression by MβCD had virtually no effect on TRPL channel activity, suggesting that in HEK293 cells unlike S2 cells, cholesterol depletion suppress directly TRPL channel activation.

## Conclusion

Cholesterol molecules are essential for the proper function of ion channels, including TRPC channels. Sequestration of cholesterol by MβCD from the plasma membrane of cells expressing TRPC channels suppressed channel activity. It is still not entirely clear what is the mechanism underlying suppression of TRPC channels activity by MβCD. Possible mechanisms include association of cholesterol to a highly flexible cholesterol binding site or perturbation of specific lipid environments. Mammalian TRPC1 and TRPC6 channels require for their activity binding to scaffold proteins located in cholesterol-rich lipid rafts. For these channels, inhibition resulting from cholesterol sequestration by MβCD possibly resulted from disruption of lipid rafts at the plasma membrane, rather than direct inhibition of a gating mechanism. Nevertheless, channel–cholesterol interactions similar to those reported for Kir channels cannot be excluded. Future availability of atomic structure of TRPC channel subfamilies nanodisks can be very useful to determine the possibility that in contrast to most other ligands, cholesterol binding to TRPC channels is highly flexible and cholesterol dynamically explores its binding site, adopting multiple poses in a “cloud,” rather than occupying a single conformation and in this way affects channel gating. For the *Drosophila* TRPL channel, where the gating mechanism of the channel is still unknown, elucidating the mechanism of cholesterol action may help solve the long-standing enigma of channel gating.

## Author Contributions

RG presented the lecture in the Israel Ion Channel and Transporters Meeting 2019, conducted a wide literature search, drafted the manuscript, and participated in the writing. MP performed the experimental part of the review and conducted part of the literature search. TB conducted part of the literature search. BK participated in the writing and drafting of the manuscript. NB wrote the section on the Computational Approach and inserted the relevant literature. IL covered the Computational Approach, searched for the relevant literature, and participated in the writing. BM initiated the review, drafted the manuscript, and wrote the paper.

## Conflict of Interest

The authors declare that the research was conducted in the absence of any commercial or financial relationships that could be construed as a potential conflict of interest.
